# Near-Infrared Light Regulation of Capture and Release of ctDNA Platforms Based on the DNA Assembly System

**DOI:** 10.3389/fbioe.2022.891727

**Published:** 2022-06-22

**Authors:** Chaihong Gong, Xiaowei Mao, Zhe Wang, Zhang Luo, Zhifan Liu, Yali Ben, Weiying Zhang, Zhenzhong Guo

**Affiliations:** ^1^ School of Life Science, Key Laboratory of Optoelectronic Chemical Materials and Devices of Ministry of Education, Jianghan University, Wuhan, China; ^2^ School of Environment and Health, Jianghan University, Wuhan, China; ^3^ School of Medicine, Jianghan University, Wuhan, China; ^4^ Hubei Province Key Laboratory of Occupational Hazard Identification and Control, Medical College, Wuhan University of Science and Technology, Wuhan, China

**Keywords:** electrochemical, NIR light-responsive, reversible, ctDNA, detection

## Abstract

Despite recent progress, a challenge remains on how to gently release and recover viable ctDNA captured on DNA probe-based devices. Here, a reusable detector was successfully manufactured for the capture and release of ctDNA by means of an UCNPs@SiO_2_-Azo/CD-probe. Biocompatible NIR light is used to excite UCNPs and convert into local UV light. Continuous irradiation induces a rapid release of the entire ctDNA-probe–CD complex from the functionalized surface *via* the trans−cis isomerization of azo units without disrupting the ctDNA-structure receptor. Specifically, these composite chips allow reloading DNA probes for reusable ctDNA detection with no obvious influence on their efficiency. The results of our study demonstrated the potential application of this platform for the quantitative detection of ctDNA and the individualized analysis of cancer patients.

## Introduction

Circulating tumor DNA (ctDNA), secreted by the tumor cells and enter into the blood, is closely related to mutations in the original tumor (Cheng et al., 2016). To date, ctDNA, deemed as a new type of redoubtable noninvasive biomarker, holds great potential application in the early diagnosis of tumors for patients (Heitzer, 2015; Qin et al., 2016; Spellman and Gray, 2014). However, detection of these ctDNA with high selectivity involves several challenges: 1) these ctDNA exist at highly rare frequency and demand specific and sensitive isolation methods and 2) profiling the heterogeneity of tumors requires different ctDNA markers to recognize various ctDNA genotypes. The heterogeneity of tumors can lead to different responses to therapy as it involves differences between ctDNA of the same type in different patients and between ctDNA within each tumor (Ma et al., 2015; Zardavas et al., 2015; Tannock and Hickman, 2016). Each organ is genetically distinct from the modified genome, and the detection of mutations in ctDNA alone cannot be traced to the organ that causes the tumor. If the detected ctDNA can be analyzed quickly, gently, and without loss, it is possible to find the diseased organs and to carry out targeted interventions in future.

As we know, most of the previous reports (Li et al., 2014; Li et al., 2015; Guo et al., 2016) concentrate on the capture and release of CTC, while the cost of CTC single cell sequencing is expensive, and it also limits the application of technology. Compared with CTC and other liquid biopsy techniques, ctDNA can reflect tumor heterogeneity more comprehensively (Abbosh et al., 2017). To date, the most commonly used technology for ctDNA testing in blood is mainly DNA sequencing and polymerase chain reaction (PCR). Although great progress has been made in ctDNA detection, the complex sample preparation and the interference from the biological environment would be problematic in further application (Zhou et al., 2016). Nowadays, a lot of evidence has suggested that nanodevices play a very important role in the successful detection of ctDNA (Nguyen and Sim, 2015). For instance, recently, Sim et al. reported a peptide nucleic acid (PNA)-based biological probe for analysis of tumor-specific genetic alterations (Nguyen and Sim, 2015; Gombos et al., 2021; Hasenleithner and Speicher, 2022; Kapeleris et al., 2022). Also, Zhou et al. developed a surface-enhanced Raman spectroscopy assay which could directly detect mutated circulating nucleic acids in patient serum (Zhou et al., 2016). However, these studies only captured ctDNA, and research on the release of ctDNA is still open wide.

Many external stimuli signals, such as enzymatic hydrolysis (Shen et al., 2013; Pinheiro et al., 2022; Hack and Bayne, 2022), pH response (Liu H et al., 2013), and temperature (Ke et al., 2015), have been used in previous literatures for the release of ctDNA in one piece. It is noteworthy that light has a great advantage over other external stimuli because it is non-contact, accurate, and controllable. One of the remarkable examples is that azobenzene, which can be readily synthesized and modified, can be reversibly photoisomerized between trans and cis configurations by alternating ultraviolet (UV) irradiation and visible light (Wang et al., 2015; Stricker et al., 2016), making it an excellent guest molecule in the supramolecular recognition system for cyclodextrin (CD) (Ueno et al., 1979; Hu et al., 2015). A supramolecular copolymer between trans-azobenzene and CD would be constructed though van der waals force and hydrophobic interaction, while cis-azobenzene conformation cannot form a host–guest complex on account of the size of host cave and guest molecular dimension mismatch. Noteworthily, ultraviolet irradiation can change the structure of DNA. In contrast, near-infrared (NIR) light holds great promise to enhance the capability for ctDNA release under a mild stimulation condition. Upconversion nanoparticles (UCNPs), which can absorb NIR light and convert it into high-energy photons in the UV, visible, and NIR regions (Liu J et al., 2013; Cui et al., 2015; Han et al., 2016), disassembled the host–guest system of azobenzene and CD due to photoisomerization on azobenzene.

**SCHEME 1:**
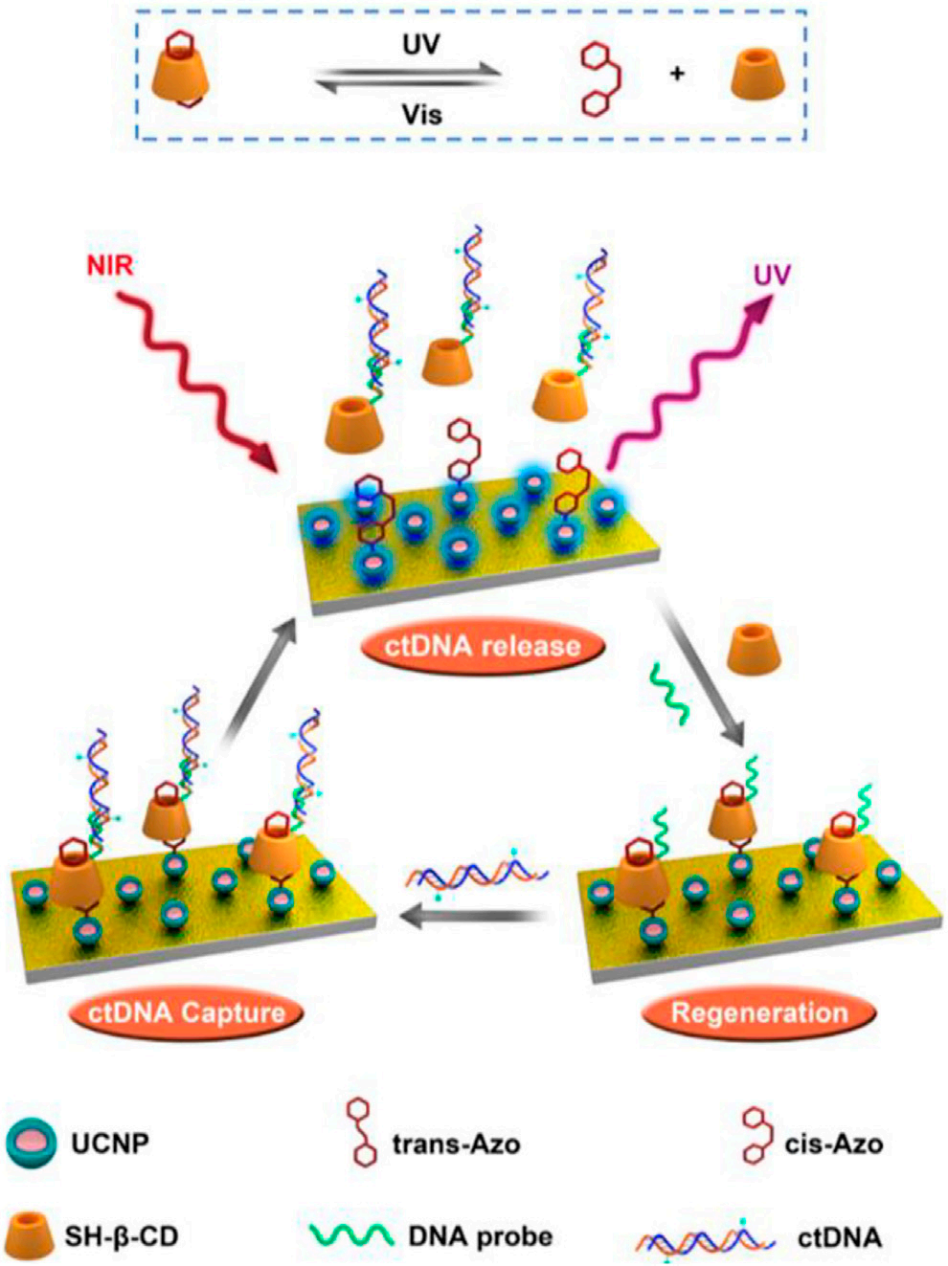
Schematic diagram of the UCNP-Azo/CD-probe as a NIR-triggered photoswitch for reversible ctDNA capture/release.

As shown in Scheme 1, here we report a smart biocompatible NIR light-responsive capture/release platform for ctDNA based on the DNA assembly system. First, UCNPs (NaYF_4_:Tm,Yb@NaYF_4_) were utilized as a core coated with an SiO_2_ shell for the construction of UCNP@SiO_2_ core–shell nanoparticles. The substrate was first modified with UCNPs@SiO_2_; then, azobenzene was functionalized with UCNPs@SiO_2_; subsequently, SH-*β*-CD was attached to the azobenzene to prepare thiol-terminated CD-modified substrates (UCNPs@SiO_2_@Azo/CD). Finally, a DNA probe was linked to SH-*β*-CD for constructing a PNA-decorated Si-CD/Azo substrate (Si-CD/Azo-probe) that could specifically capture ctDNA (PIK3CA E542K) through a host–guest recognition effect between azobenzene and *β*-CD. This UCNPs@SiO_2_@Azo/CD-probe substrate can specifically capture ctDNA and release it via near-infrared light (NIR). After NIR light irradiation, the azobenzene converted from trans- to cis-isomers, leading to the *β*-CD being unable to encapsulate cis-azobenzene, thus releasing the as-captured ctDNA. By visible light irradiation, the UCNPs@SiO_2_@Azo substrate then recovers the function for binding *β*-CD to construct the UCNPs@SiO_2_@Azo/CD-probe. The reversibility of the differential pulse voltammetric (DPV) measured with the addition of *β*-CD and ctDNA could be repeated for several cycles. As a result, we constructed a smart system by using a UCNPs@SiO_2_@Azo/CD-probe substrate with NIR light-responsive circulative capture and release of ctDNA in biological samples.

## Results and Discussion

**SCHEME 2:**
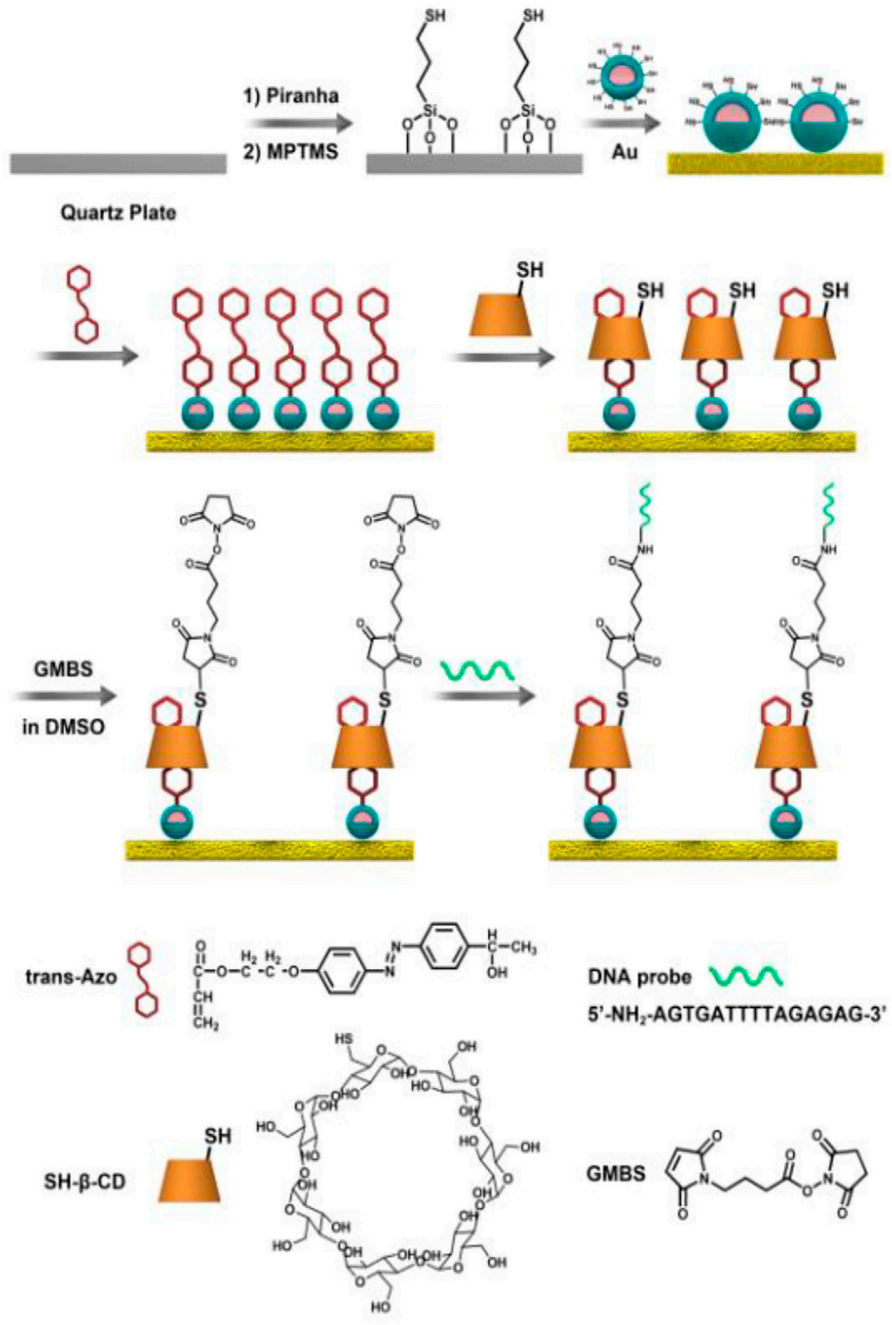
Modification of the UCNPs@SiO_2_@Azo/CD-probe on the substrate.

**FIGURE 1:**
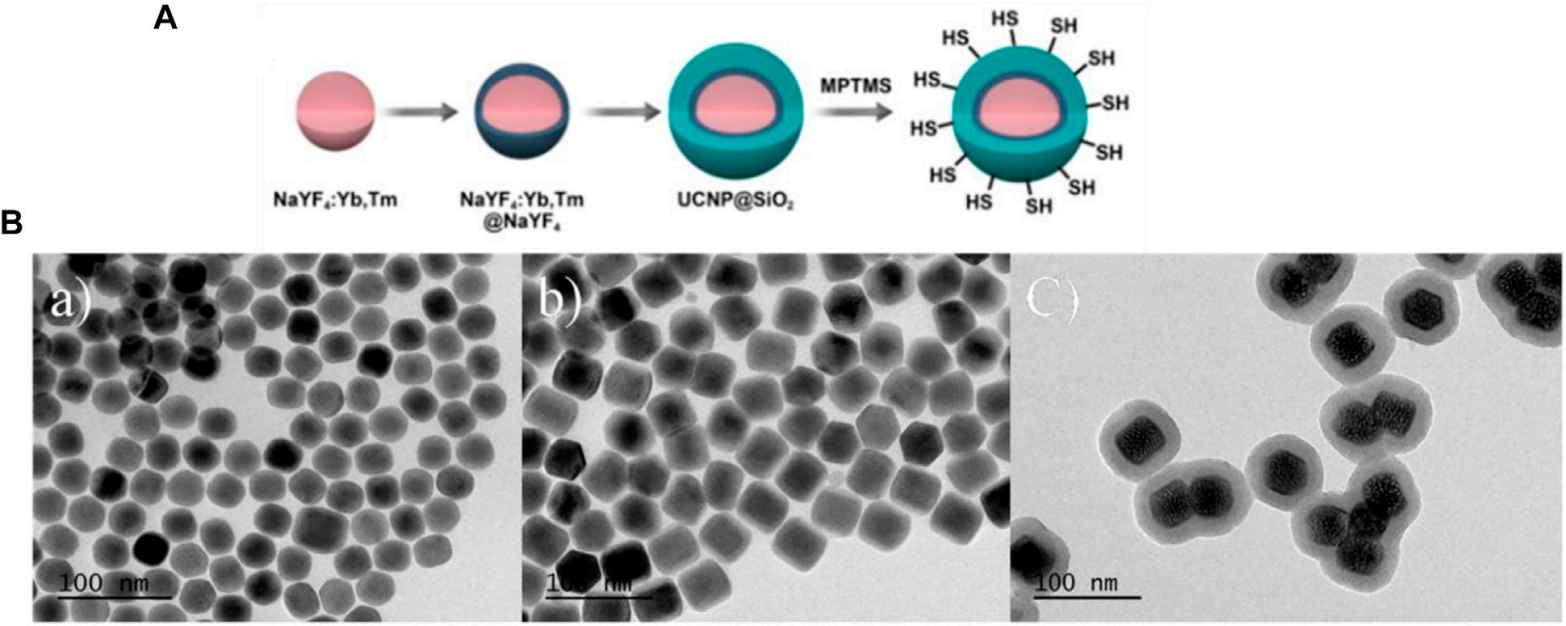
**(A)** Synthesis procedures of UCNPs, **(B)**TEM images of core **(a)**, core–shell **(b)** nanoparticles for NaYF4:Tm/Yb and NaYF4:Tm/Yb@NaYF4 UCNPs, respectively. UCNPs@SiO_2_ NPs **(c)**.

For the construction of a reusable surface for the capture and release of ctDNA, a surface decorated with the UCNPs@SiO_2_@Azo/CD-probe was fabricated (Scheme 2). As shown in Figure 1A, UCNPs (NaYF_4_:Tm,Yb@NaYF_4_) were applied as a core coated with an SiO_2_ shell to obtain UCNP@SiO_2_ core–shell nanoparticles. As shown in Figures 1Ba,b, the UCNPs are nearly monodisperse particles. The size of UCNPs is 30 and 40 nm for the core and core–shell mode, respectively. The core–shell UCNPs are highly crystalline and hexagonal in phase, as confirmed by powder X-ray diffraction (Supplementary Figure S1). However, UCNP@SiO_2_ NPs with about 10 nm silica shell were confirmed by TEM imaging (Figure 1Bc). FT-IR measurement also verified the silica functional procedure (Supplementary Figure S2, Supporting Information). As displayed in Supplementary Figure S3, we applied a self-assembly process for the integration of mono-layered nanofilms of UCNPs on a quartz slide (1 cm × 1 cm).

**FIGURE 2:**
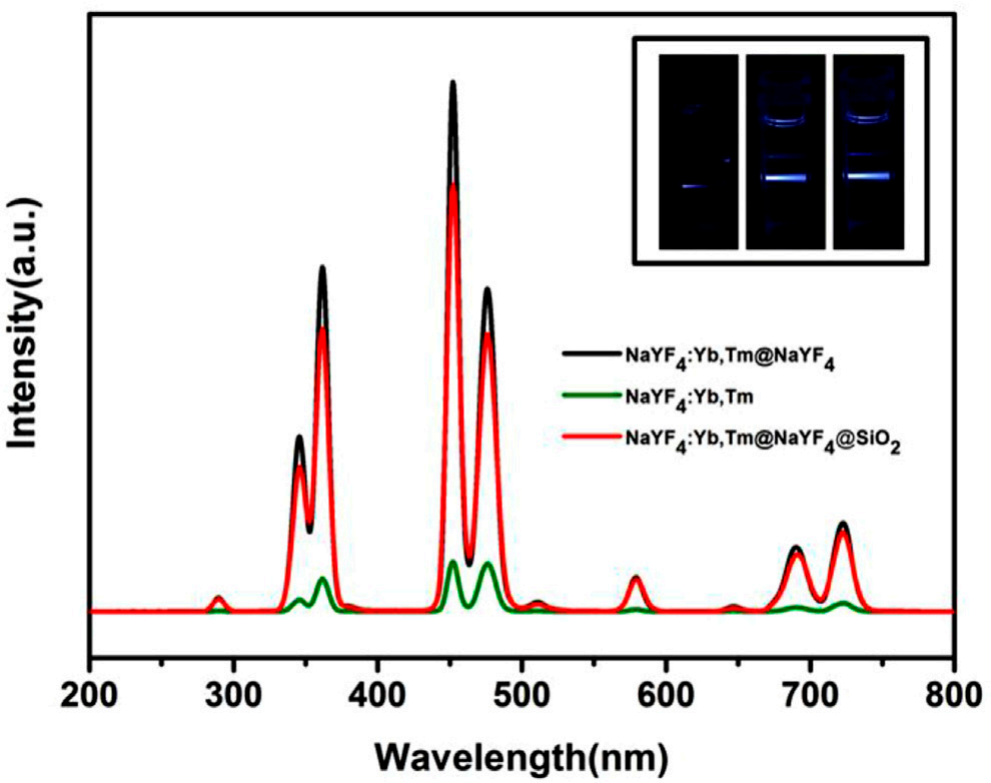
Luminescence analysis of NaYF_4_:Tm,Yb (green), NaYF_4_:Tm,Yb@NaYF_4_@SiO_2_ (red), and NaYF_4_:Tm,Yb@NaYF_4_ (black). The corresponding fluorescent photographs are shown in the inset images from the left to right. All samples at 1 mg/ml were used in the experiment.

NaYF_4_:Tm,Yb, recently reported as a new type of upconversion fluorescent nanoparticle, with photoluminescence (PL) property under NIR laser excitation (*λ* = 980 nm, 6.5 W/cm^2^), was prepared and characterized by fluorescence measurement as shown in Figure 2. All the upconversion fluorescent nanoparticles exhibit good luminescent properties with emission peaks at about 365, 452, and 474 nm under excitation at 980 nm laser. It is noteworthy that after chemical modification of the NaYF4 shell, NaYF_4_:Tm,Yb@NaYF_4_ nanoparticles show stronger fluorescence intensity than NaYF_4_:Tm. The fluorescence intensity of NaYF_4_:Tm,Yb@NaYF_4_@SiO_2_ decreased after being modified by the silicon shell, which is in agreement with previous reports (Wang et al., 2015). These manifestations were also confirmed by a digital photo (Figure 2 inset).

**FIGURE 3:**
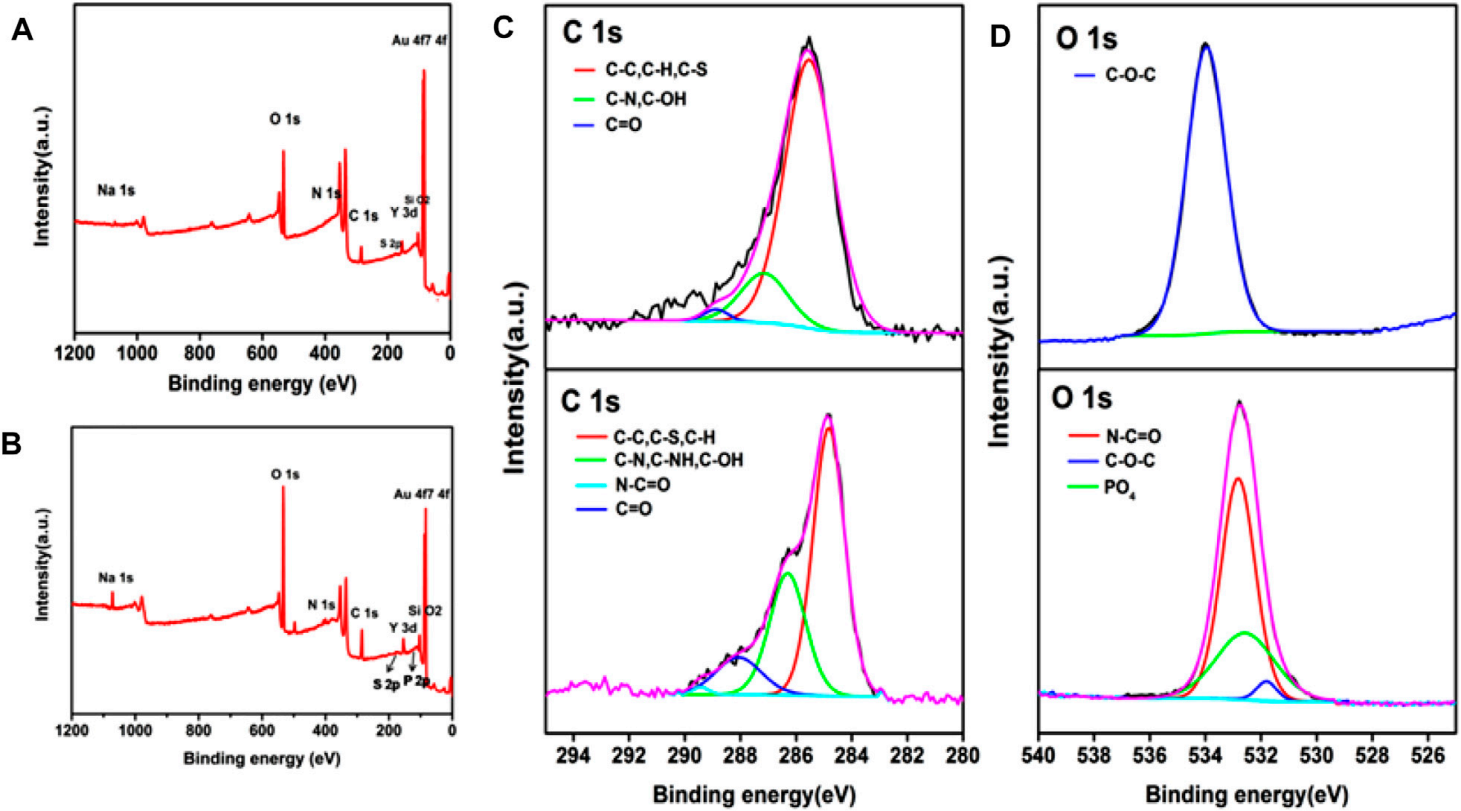
XPS wide spectra of **(A)** UCNPs@SiO_2_@Azo and **(B)** UCNPs@SiO_2_@Azo/CD-probe. XPS C1s **(C)** and O1s **(D)** core-level spectra of UCNPs@SiO_2_@Azo (top) and UCNPs@SiO_2_@Azo/CD-probe (bottom) surfaces, respectively.

*β*-SH-CD was prepared for grafting to UCNPs@SiO_2_@Azo by means of host–guest self-assembly. Subsequently, a specific DNA probe, used as a ctDNA capture agent, was led to the *β*-SH-CD using chemical coupling agents to obtain the DNA probe with *β*-CD-thiol exposed outside (UCNPs@SiO_2_@Azo/CD-probe). Therefore, specific catching of ctDNA can be realized by interactions between azobenzene and the CD host–guest supramolecular system. The azobenzene, well known for its correspondent transform that could participate in this CD supramolecular self-assembly recognition system, was prepared in accordance with previous literature (Wu et al., 2012). A red shift of about 20 nm in the plasmonic peak after modification of Azo is presented in Supplementary Figure S4. In addition, GMBS was introduced onto the as-formed surface, which acted as a coupling agent for a DNA probe for establishment of the ctDNA analytical surface. X-ray photoelectron spectroscopy (XPS) is a classical characterization method that provides critical chemical bonding information for DNA modification procedures (Saoudi et al., 2004). The chemical compositions of the as-prepared UCNPs@SiO_2_@Azo substrate revealed by XPS measurement are shown in Figure 3A. It is obvious that the binding energies at 168.1 and 134.3 eV appeared and related to S 2p of *β*-CD and P 2p of DNA sequence signals, respectively, showing that the DNA probe has been successfully coupled to the UCNPs@SiO_2_@Azo/CD-probe (Figure 3B). The carbon (C) and oxygen (O) spectra in the XPS measurement were also confirmed by the DNA probe attached to the UCNPs@SiO_2_@Azo/CD surface, as shown in Figures 3C,D. Comparing UCNPs@SiO_2_@Azo with UCNPs@SiO_2_@Azo/CD-probe surfaces in the C1s XPS spectrum, two shoulder peaks at 289.4 eV were observed due to the N−C=O chemical groups derived from the nucleic bases (Saoudi et al., 2004; Chandra et al., 2011). Additionally, three peaks at 532.9, 531.8, and 532.5 eV in O1s characterization correspond to N−C=O, C−O−C, and PO_4_ bonding, respectively (Qi et al., 2015). The peaks at 532.9, 531.8, and 532.5 eV were assigned to the oxygen in the nucleobase, the ether bond of *β*-CD, and the backbone phosphate group of the DNA sequence, respectively. In conclusion, the results discussed clearly demonstrate that the DNA probe was evidently grafted onto the UCNPs@SiO_2_@Azo.

**FIGURE 4:**
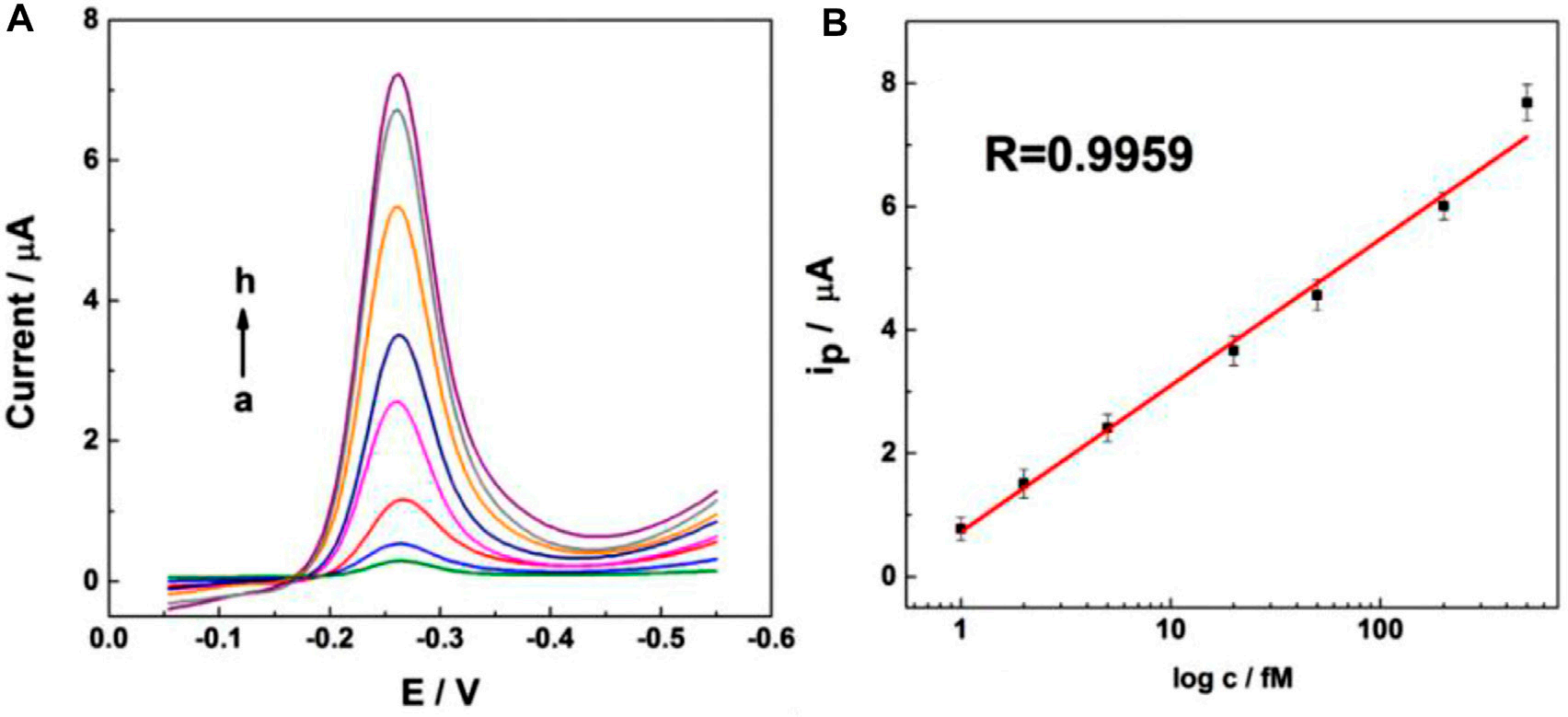
Differential pulse voltammetric (DPV) response for UCNPs@SiO_2_@Azo/Au electrode incubated with different concentrations of ctDNA in 0.1 M pH 7.4 phosphate buffer; pulse period, 0.2 s; and amplitude, 50 mV.

To confirm the ctDNA capturing ability of this UCNPs@SiO_2_@Azo/CD-probe, 30 min was chosen as the capture time by means of fluorescein diacetate (Gene Finder) as a model to trace the capture process directly. To the whole substrate, 40 μL ctDNA (3.91 nM) in PBS was added. As shown in Supplementary Figure S5A, the substrate was stained bright green fluorescent and exhibited high-efficiency capturing activity after 30 min of incubation. For the sake of ctDNA detection sensitivity, the chip was challenged with different concentrations of ctDNA. Figure 4A illustrates the DPV response of the UCNPs@SiO_2_@Azo/CD-probe for different concentrations of ctDNA (1, 2, 5, 20, 50, 200, and 500 fM). The current response of the oxidation (*i*pa) process shows a good linear relationship with the analyte concentration (1–500 fM) for ctDNA (Figure 4B). The detection limit was 1 nM as calculated based on the 3σ IUPAC criteria for three times the DPV response of ctDNA in the UCNPs@SiO_2_@Azo/CD-probe, demonstrating its good sensitivity and stability. This result shows this probe is more sensitive than the carbon nanotube-modified GC electrode (37 μM for DA). After electrochemical detection, the substrate was immersed in pH 7.4 PBS solution and exposed to 6.5 W/cm^2^ NIR light for 10 min (2 min break after 2 min irradiation). Then the solution is amplified by PCR, and the amplified PCR product is characterized. As displayed in Supplementary Figure S5B, the results of the PCR measurement clearly demonstrates that NIR irradiation induced a rapid release of ctDNA from the substrate. Therefore, the presence of fluorescent bands in the gel indicates the release of ctDNA was successful.

## Conclusion

In summary, we have successfully fabricated an UCNPs@SiO_2_@Azo/CD-probe chip with switchable NIR. First, a DNA probe was utilized to selectively identify the tumor ctDNA. Then, UCNPs were used to convert the NIR light into local UV light for ctDNA capture or release by means of supermolecule host–guest assembling interaction between azobenzene and *β*-CD, which has created opportunities for precision cancer management in the future. This UCNPs@SiO_2_@Azo/CD-probe chip can be easily regenerated for the next cycles. Therefore, we designed an UCNP-based chip that could capture ctDNA with high sensitivity and selectivity. This study proves the significance of controllable ctDNA on the chip. The effective release of ctDNA provides a facile and effective strategy for nondestructive release, and can be used for the dynamic study of cancer therapy in the early stage.

## Data Availability

The original contributions presented in the study are included in the article/Supplementary Material; further inquiries can be directed to the corresponding author.
