# Supplementary material for: Near-Infrared Light Regulation of Capture and Release of ctDNA Platforms Based on the DNA Assembly System
Source: Front Bioeng Biotechnol. 2022 Jun 22;10:891727. doi: 10.3389/fbioe.2022.891727 (PMC9272789; doi:10.3389/fbioe.2022.891727)
Supplement: Supplementary file 1 [file DataSheet1.docx]

**Supporting information**

Near infrared light regulation of capture and release of ctDNA platform based on DNA assembly system

*Chaihong Gong^a‡^, Xiaowei Mao^c‡^, Zhe Wang^b^, Zhang Luo^a^, Zhifan Liu^a^, Yali Ben^b^*, Weiying Zhang^a^*, Zhenzhong Guo^d^*

^a^Key Laboratory of Optoelectronic Chemical Materials and Devices of Ministry of Education, Jianghan University, Wuhan 430056, PR China

^b^School of medicine, Jianghan University, Wuhan 430065, PR China

^c^School of Environment and Health, Jianghan University, Wuhan 430056, P. R. China

^d^Hubei Province Key Laboratory of Occupational Hazard Identification and Control, Medical College, Wuhan University of Science and Technology, Wuhan 430065, PR China

*To whom correspondence should be addressed. Email: zwy2428@163.com; Telephone: (+)86 13098885424

**Contents**

1. Materials and Reagents*....…………...**………………**…………..………..………………S3*
2. Experimental section details*………………**……………………..………..………………S3*
3. XRD patterns of the β-NaYF4:Tm,Yb@β-NaYF4..……………...………………S7
4. FITR spectra of the bare UCNPs and UCNPs@SiO_2_……………...………………S8
5. SEM images of UCNPs@SiO_2_ NPs………...……………………..…….………..…S9
6. UV−vis spectra of the azo, UCNPs@SiO2-SH and Azo, UCNPs@SiO2@Azo…...S10

7.  The DPV response for ctDNA UCNPs@SiO_2_@Azo /Au electrode………………...S11

1. **Experimental**

**1.1** *Materials and Reagents.*

The nucleotides sequences used in this study as following: DNA probe for E542K: 5’-NH_2_-AGT GAT TTT AGA GAG; ctDNA: 5’-CAmCGA GAT CCT CTC TCT AAA ATC ACT GAG CAG GAG AAA GAT TTT CTA TGG AGT CAC AGA CAC TAT TGTG and 3’- GTG CTC TAG GAG AGA GAT TTT AGT GAT TCG TCC TCT TTC TAA AAG ATA CCT CAG TGT CTG TGA TAA CAC were purchased from Wuhan Genecreate Biological Engineering Co. Ltd., (Wuhan, China). oleic acid (90%), Yb(C_2_H_3_O_2_)3·4H_2_O (99.99%), Y(C_2_H_3_O_2_) 3·xH_2_O (99.99%), Tm(C_2_H_3_O_2_) 3·xH_2_O(99.99%), ammonia aqueous solution (25%) and Tetraethylorthosilicate (TEOS) were all purchased from Shanghai Aladin Co., Ltd. (China). Mono(6-mercapto-6-deoxy)-β-cyclodextrin (SH-β-CD) was supplied by Shandong Binzhou Zhiyuan Bio-Technology Co., Ltd. (China). N-γ-maleimidobutyryloxy succinimide ester (4-maleimidobutyric acid N-hydrosuccinimide, GMBS), CO-520 and 3-Mercaptopropyl-trimethoxysilane (MPTMS, 95%) were purchased from Sigma-Aldrich. NaOH, NH_4_F, ethanol, cyclohexane were purchased from China National Medicine Corporation Ltd. Other reagents were commercially available and were of analytical grade. All solutions were prepared using ultrapure water (≥18 MΩ, Millipore). Azo was prepared as described in previous works.[24]

**1.2** *Instrumentations.*

TEM micrographs were taken under a Tecnai G2 F20 S-TWIN (FEI, USA). Scanning electron microscopy (SEM, HITACHI SU8010) was applied to characterize the morphology in this paper. The UV-vis absorption spectra were recorded on a UV-2550 spectrophotometer (Shimadzu, Japan). X-ray diffraction (XRD) patterns were performed on a PANalytical XRD system (X'Pert Powder) with Cu Ka radiation (l=1.54056 Å). X-ray photoelectron spectra (XPS) was measured with a Thermo Escalab250 XI spectrophotometer. Infrared spectra were recorded by a Perkin Elmer 782 series Fourier transform infrared (FT-IR) spectrophotometer (American). Upconversion fluorescence emission spectra were recorded on Perkin Elmer LS55 fluorescence spectrometer (American), equipped with an external adjustable 980 nm NIR laser. SWV measurements were carried out on a CHI 660C workstation (CH Instruments Inc., Shanghai, China).

**1.3** *Synthesis of* ***NaYF_4_: Tm, Yb and NaYF_4_: Tm, Yb@NaYF_4_*** *nanocrystals.*

Lanthanide-doped NaYF_4_ nanocrystals with monodisperse nanoparticle were prepared as described elsewhere.[25] In a typical process, Y(C_2_H_3_O_2_) 3·xH_2_O (482.3 mg, 1.59 mmol), Yb(C_2_H_3_O_2_) 3·4H_2_O (154.9 mg, 0.4 mmol), and Tm(C_2_H_3_O_2_) 3·xH_2_O (3.83 mg, 0.01 mmol) in deionized water were added to a 100 mL flask containing 15 mL oleic acid and 30 mL 1-octadecene. After stirring at room temperature for 30 min, the mixture was slowly heated to 120 °C to get rid of water under nitrogen atmosphere, and maintained at 156 °C for about 1 h until a homogeneous transparent yellow solution was obtained. The system was then cooled down to room temperature with the flowing of nitrogen. Then 10 mL methanol solution of NH_4_F (296.3 mg, 8 mmol) and NaOH (200 mg, 5 mmol) was added and the solution was stirred at room temperature for 2 h. After methanol evaporated, the solution was heated to 310 °C and kept for 1.5 h before it was cooled down to room temperature. The mixture was first precipitated by the addition of 20 mL ethanol, and collected by centrifugation at 8000 r/min for 10 min. Product was re-dispersed with 5 mL cyclohexane and precipitated by adding 15 mL ethanol, then collected by the same centrifugation. After four times’ washing, the final product was re-dispersed in 20 mL cyclohexane.

For the synthesis of NaYF_4_:Tm,Yb@NaYF_4_ nanocrystal, about 1.0 mmol NaYF_4_:Tm,Yb was firstly prepared using the similar procedures as mentioned above. Then, 800 μmol Y(C_2_H_3_O_2_)3·xH_2_O in water solution was added in to a 100 mL flask containing 15 mL oleic acid and 30 mL 1-octadecene. The solution was stirred at room temperature for 30 min. Then the mixture was slowly heated to 120 °C to get rid of water under nitrogen atmosphere, and maintained at 156 °C for about 1 h until a homogeneous transparent yellow solution was obtained. The system was then cooled down to room temperature with the flowing of nitrogen. Then, 5 mL pre-prepared NaYF_4_:Tm, Yb core (dispersed in cyclohexane) was added and kept for another 30 min before heated to 80 °C to remove cyclohexane. Then, 10 mL methanol solution of NH4F (1 mmol) and NaOH (1.685 mmol) was added and the solution was stirred at room temperature for 2 h. After methanol evaporated, the solution was heated to 310 °C and kept for 1.5 h before it was cooled down to room temperature. The same washing steps were followed and sample was re-dispersed in 20 mL cyclohexane.

**1.4** *Synthesis of* ***UCNPs@SiO2-SH***

To make the UCNPs more stable and easier for surface modification, the oleate-capped UCNPs were further coated with a thin amorphous layer of silica by the reverse microemulsion method according to literature procedures. [26] Briefly, 500 μL of CO-520, 8 mL of cyclohexane, and 2 mL of UCNP solution were mixed and stirred for 10 min. 100 μL of 25 wt % ammonia was then added and the container was sealed and sonicated for 20 min until a transparent emulsion was formed. 80 μL of TEOS was then added into the solution. After stirring for a few minutes, 200 μL of 3-Mercaptopropyl-trimethoxysilane (MPTMS) was then added dropwise into the resultant mixture to modify the silica surface with sulfhydryl groups. The solution was stirred for 48 h at a speed of 600 rpm.The product were centrifuged and washed with ethanol twice, and redispersed in 6 mL of water.

***1.5*** *Modified the* ***UCNPs@SiO2-SH*** *on the surface of substrate*

A quartz substrate was immersed into a fresh piranha solution (30% H2O2/98% H2SO4, v/v =1:3; Caution: Piranha solution is a very aggressive, corrosive solution, and appropriate safety precautions should be utilized, including the use of acid-resistant gloves and adequate shielding) and heated until no bubbles were released. The surface of the chip was modified with 4% (v/v) 3-mercaptopropyl-trimethoxysilane (MPTMS) in ethanol at room temperature for overnight, and then was dehydrated at 60 °C for 1 h to obtain the sulfhydryl -silanized quartz slide. [27] Then, the chip is placed in the ion sputtering instrument, it is sprayed with gold and removed after 30s. Next, the substrate (1 cm×1 cm) into the UCNPs@SiO_2_-SH solution, and then agitated them on an air bath constant temperature oscillator at 25 °C overnight, followed by a washing with ethanol. Finally, then was dehydrated at 60 °C for 1 h.

***1.6*** *Preparation of the* ***UCNPs@SiO2-Azo*** *linker surface*

The substrates was combined with Azo（2 mg/mL） and 0.01 g of AIBN in isopropanol. The solution was then heated at 60°C on an air bath constant temperature oscillator at 60°C overnight, followed by a washing with ethanol. Finally, the substrate was dried with nitrogen.[28]

***1.7*** *Synthesis of DNA probe-Modified* ***UCNPs@SiO_2_-Azo*** *Substrates (****UCNPs@SiO_2_-Azo/CD-probe****)*

The substrate into the SH-β-CD solution (2 mg/mL), and then agitated them on an air bath constant temperature oscillator at 25 °C overnight, followed by a washing with ultrapure water. Finally, the substrate was dried with nitrogen. Then, the substrate was rinsed with DMSO and treated with a coupling agent GMBS (0.25 × 10^−3^ M in DMSO) for 30 min. Next, the substrate was treated with PBS solution of DNA probe (1 mM) for 2 h and then washed with PBS to remove excess DNA probe. The substrates were finally dried with a flow of ultrapure N_2_ and stored at 4°C. [29]

***1.8*** *ctDNA Capture and Release Assay.*

**ctDNA Capture:** For ctDNA capture, the probe-immobilized substrate, ctDNA suspensions (50 fM, 500 fM,5000 fM) were added to each culture plate, leading to the ctDNA attached on the quartz substrate. The substrates were incubated 30 min in. After ctDNA capture, the substrate was detected by electrochemical workstation.

**ctDNA Release:** After capture, ctDNA were examined for NIR triggered release. The substrate was immersed in pH 7.4 PBS solution containing the UCNPs. After exposing to NIR laser at the power density of 6.5 W/cm^2^ for 10 min (2 min break after 2 min irradiation to avoid heating effect on ctDNA), the substrate was gently washing with PBS for 15 s to remove the released ctDNA. The released ctDNA was amplified by PCR and detected by gel electrophoresis.


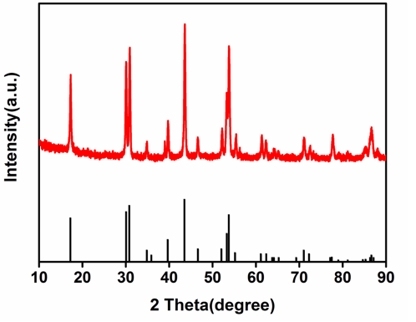


***Figure S1.*** Powder X-ray diffraction (XRD) patterns of the β-NaYF_4_: Tm, Yb@β-NaYF_4_ core/shell UCNPs ( JCPDS no. 16-0334).


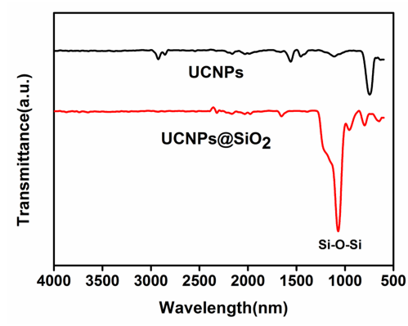


***Figure S2.*** FITR spectra of the bare UCNPs (black line), UCNPs@SiO_2_ (red line). For the bare UCNPs capped with a layer of oleic acid, the 2924 and 2856 cm^-1^ band were the stretching vibration of methylene (CH_2_) in the oleic acid molecules and the bands at 1559 and 1455 cm^-1^ were due to the stretching band of the carboxylic group. After coating of silica shell, new strong bands attributed to the stretching vibration and deformation vibrations of the Si-O bond could be seen clearly at 1069 and 797 cm^-1^, respectively. These suggested that the nanoparticles coated the SiO_2_ successfully.


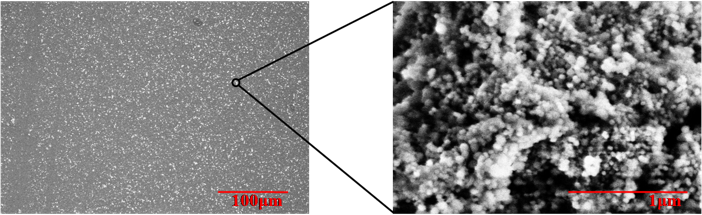


***Figure S3.***SEM images of UCNPs@SiO_2_ NPs on the surface of substrate.


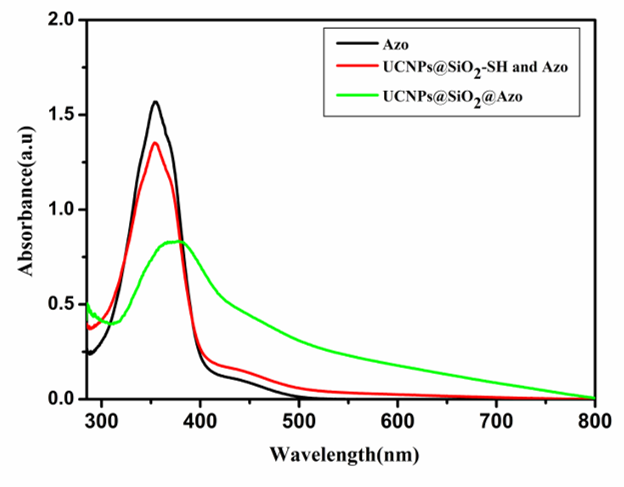


***Figure S4.*** UV-vis spectra of the azo(black), UCNPs@SiO_2_-SH and Azo(red) and UCNPs@SiO_2_@Azo(green).


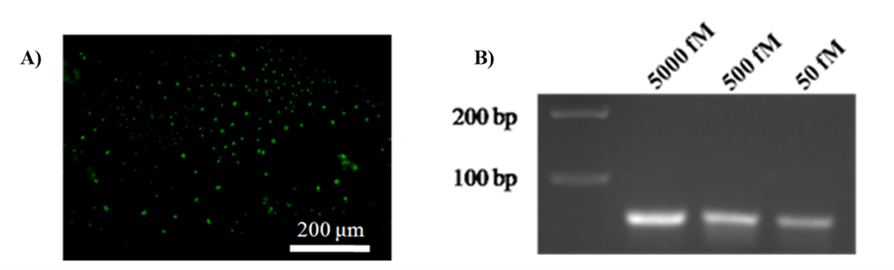


***Figure S5.*** A) Fluorescence images of ctDNA captured on the substrate; B) PCR analysis of the ctDNA release from the substrate.
